# Supplementary material for: Structural Bioinformatics Applied to Acetylcholinesterase Enzyme Inhibition
Source: Int J Mol Sci. 2025 Apr 17;26(8):3781. doi: 10.3390/ijms26083781 (PMC12028328; doi:10.3390/ijms26083781)
Supplement: Supplementary file 1 [file ijms-26-03781-s001.zip › ijms-3516681-supplementary.pdf]

Supplementary Material

Table S1. Acetylcholinesterase (AChE) as target in docking studies.

PDB	Ligand	Affinity energy (kcal/mol)	Software	Cite
4M0E	Amarosterol A	-10	Vina	[19]
	Hinokinin	-9.8		
	β-sitosterol	-9.6		
	Stigmasterol	-9.5		
	Ellagic acid	-9.2		
	Eserine	-8.2		
1EVE	Donepezil	-9.81	Glide	[43]
	Flavonol glucoronide querciturone	-13.43		
	Phenolic glycoside	-11.51		
	luteolin 30 ,40 -di-O-glucoside	-11.38		
	kaempherol-3-O-rhamoside	-11.27		
	2-linoleoyl glycerol	-10		
	daucosterol	-10.11		
4EY7	(Z)-ethyl-2-(3-oxo-1,3- diphenylprop-1-enylamino) acetate	-10.7	AutoDock4	[46]
	Ethyl 3,5-diphenyl-1H-pyrrole-2-carboxylate	-10		
3I6M	C15H11C1O	-8.4	AutoDock4	[47]
	C17H15NO3	-8.29		
	C16H13C1O2	-8.14		
	C15H11BrO2	-8.55		
	C15H10BrNO3	-8.16		
5FPQ	Rivastigmine	-7.5		
5HF5	Rivastigmine	-7.5		
6O4X	Rivastigmine	-7.4		
4EY4	Rivastigmine	-7.3		
6WVQ	Rivastigmine	-7.3		
6NTO	Rivastigmine	-7.2		
6NTL	Rivastigmine	-7.1		
6WUZ	Rivastigmine	-7.1		
4MOE	Rivastigmine	-6.9		
6CQZ	Rivastigmine	-6.9		

6U37	Rivastigmine	-6.9		
4M0E	Ondansetron	-6.364	Schrödinger suite	[49]
	Tacrine	-6.563		
	Rivastigmine	-5.616		
6WVO	7a	-6.2	Vina	[50]
	7b	-6.6		
	7c	-7		
	7d	-7.4		
	7e	-7.2		
	7f	-6.7		
	7g	-9.2		
	7h	-9		
4EY7	2-((2-(4-Benzylcyclohexyl)ethylimino)methyl)-5-benzyloxy-1-methylpyridin-4(1H)-one	-9.9		[51]
	5-(Benzyloxy)-2-((1-benzylpiperidin-4-ylimino)methyl)-1-methylpyridin-4(1H)-one	-11.1		
	2-(((4-Benzylcyclohexyl)yridineno)methyl)-5-(benzyloxy)-1-methylpyridin-4(1H)-one	-10.3		
	2-((1-(4-Methoxyphenethyl)piperidin-4-ylimino)methyl)-5-(benzyloxy)-1-methylpyridin-4(1H)-one	-10.5		
	5-(Benzyloxy)-2-((1-isopropylpiperidin-4-ylimino)methyl)-1-methylpyridin-4(1H)-one	-10.5		
	5-(Benzyloxy)-1-methyl-2-((phenethylimino)methyl)yridine-4(1H)-one	-10.3		
	Donepezil (Reference drug)	-10.5		
1ACL	N-((5-chloro-1H-indol-2-yl)methyl)-4-fluorobenzenesulfonamide	-6.4028	MOE	[52]
	N-((5-chloro-1H-indol-2-yl)methyl)-2-fluorobenzenesulfonamide	-6.4638		

	N-((5-chloro-1H-indol-2-yl)methyl)-4-(trifluoromethyl)benzenesulfonamide	-6.0869		
	Donepezil	-6.3226		
1W6R	3-Methoxyquercetin	-8.43	AutoDock4	[53]
1W6R	Quercetin	-8.38		
1W6R	Galantamin	-9.63		
1C2B	Arcabose (control)	-4.8	Vina	[54]
1C2B	Kaempferol	-8.6		
1C2B	Quercetin	-6.9		
1C2B	Emodin	-7.7		
1C2B	Chrysophanol	-3.8		
1C2B	Donepezil (Reference drug)	-11.7		
1W6R	Compound 1	-12.58	AutoDock4	[55]
1W6R	Compound 2	-10.6		
1W6R	Compound 3	-10.52		
1W6R	Compound 4	-7.94		
1W6R	Compound 5	-7.25		
1W6R	Physostigmine	-8.81		
1W6R	Galanthamine	-8.68		
4EY7	Co-CL	-12.1	Vina	[56]
	Clozapine	-9.1		
1DX6	AC1	-25.83	Vina	[57]
	AC2	-26.03		
	AC3	-27.67		
	AC4	-24.49		
	AC5	-17.71		
	AC6	-25.45		
	AC7	-17.94		
	AC8	-26.27		
	AC9	-27.8		
	AC10	-27.3		
	AC11	-25.33		
	AC12	-22.15		
	AC13	-23.3		
	Galantamina	-28.53		
1EVE	Donepezil	-9.81	Glide	[58]
	2-linoleoyl glycerol	-10		
	daucosterol	-10.11		

	kaempherol-3-O-rhamoside	-11.27		
	luteolin 30 ,40 -di-O-glucoside	-11.38		
	Phenolic glycoside	-11.51		
	Flavonol glucoronide querciturone	-13.43		
4EY7	Sanguinine	-11.5	Vina	[58]
	Huperzine A	-10.9		
	Crinine	-10.5		
	Galantimine (Reference drug)	-10.4		
	Donepezil (Reference drug)	-11.7		
6O4W	Chrysin	-11.148	Schrödinger suite	[58]
	Convolvine	-13.191		
	Galangin	-10.28		
	Isoliquiritigenin	-10.322		
	Naringenin	-11.56		
	Palmatoside G	-14.676		
	Quercetin	-12.397		
	Rugin	-17.023		
4EY5	Huperzine-4C-Naringenin	-14.1	Vina	[59]
	Naringenin-4C-Galantamine	-14		
	Huperzine-4C-Galantamine	-13.4		
	Huperzine-5C-Carvone	-13		
	Yohimbine-5C-Carvone	-13		
	Galantamine-4C-Carvone	-12.9		
	Sanguinarine-4C-Carvone	-12.7		
	Berberine-4C-Carvone	-11.8		
	Chelerythrine-4C-Carvone	-11.8		
	Berberastine-4C-Carvone	-11.6		
	Akuammicine-4C-Carvone	-11.1		
	Donepezil (control)	-10.5		
	Galantamine (control)	-7.7		
1C2B	Galanthamine	-7.9	Blind docking	[60]
	Buphanidrine	-8.3		
1EVE	N,N -dimethyl-2-phenylbenzodoxazole-6-carboxamide	-6.65	Schrödinger suite	[61]
	N,N-diethyl-2-phenylbenzodoxazole-6- carboxamide	-6.58		

	2-Phenyl-N,Ndipropylbenzodoxazole-6-carboxamide	-6.27		
	(2-Phenylbenzod oxazol-6-yl)(pyrrolidin-1-yl) methanone	-6.48		
	(2-Phenylbenzod oxazol-6-yl)(piperidin-1-yl) methanone	-6.83		
	Morpholino(2-phenylbenzod oxazol-6-yl) methanone	-6.79		
	2-(1,1′-Biphenyl-4-yl)-N,N-dimethylbenzod oxazole-6-carboxamide2-(1,1′-Biphenyl-4-yl)-N,N-diethylbenzodoxazole6-carboxamide	-6.61		
	2-(1,1′-Biphenyl-4-yl)-N,N-dipropylbenzod oxazole-6-carboxamide	-6.65		
	(2-(1,1′-Biphenyl-4-yl)benzodoxazol-6-yl) (pyrrolidin-1-yl)methanone	-6.49		
	(2-(1,1′-Biphenyl-4-yl)benzodoxazol-6-yl) (piperidin-1-yl)methanone	-6.79		
	(2-(1,1′-Biphenyl-4-yl)benzodoxazol-6-yl) (morpholino)methanone	-6.97		
	(2-(1,1′-Biphenyl-4-yl)benzodoxazol-6-yl) (morpholino)methanone	-6.93		
	N,Ndimethyl-2-(naphthalen-1-yl)benzodoxazole6-carboxamide	-6.58		
	N,Ndiethyl-2-(naphthalen-1-yl)benzodoxazole-6- carboxamide	-6.24		
	2-(Naphthalen-1-yl)-N,Ndipropylbenzodoxazole-6-carboxamide	-6.07		
	(2-(Naphthalen-1-yl)benzodoxazol-6-yl)(pyrrolidin1-yl)methanone	-6.41		
	(2-(Naphthalen-1-yl)benzodoxazol-6-yl)(piperidin1-yl)methanone	-6.78		
	Morpholino(2-(naphthalen-1-yl)benzodoxazol-6- yl)methanone	-6.3		
	N,Ndimethyl-2-(pyridin-4-yl)benzodoxazole-6- carboxamide	-6.23		

	N,Ndiethyl-2-(pyridin-4-yl)benzodoxazole-6- carboxamide	-6.42		
	N,Ndipropyl-2-(pyridin-4-yl)benzodoxazole-6- carboxamide	-6.35		
	(2-(Pyridin-4-yl)benzodoxazol-6-yl)(pyrrolidin-1-yl) methanone	-6.41		
	Piperidin-1-yl(2-(pyridin-4-yl)benzodoxazol-6-yl) methanone	-6.76		
	Morpholino(2-(pyridin-4-yl)benzodoxazol-6-yl) methanone	-6.79		
	N,N-dimethyl-2-(1 H-pyrrol-2-yl)benzodoxazole-6- carboxamide	-6.59		
	N,N-dipropyl-2-(1 H-pyrrol-2-yl)benzodoxazole-6- carboxamide	-6.45		
	(2-(1 H-pyrrol-2-yl)benzodoxazol-6-yl)(pyrrolidin1-yl)methanone	-6.8		
	(2-(1 H-pyrrol-2-yl)benzodoxazol-6-yl)(piperidin-1- yl)methanone	-6.98		
	(2-(1 H-pyrrol-2-yl)benzodoxazol-6-yl) (morpholino)methanone	-7.29		
	2-(Furan-2-yl)-N,N-dimethylbenzodoxazole-6-carboxamide	-6.26		
	N,N-diethyl-2-(furan-2-yl)benzodoxazole-6- carboxamide	-6.21		
	2-(Furan-2-yl)-N,N-dipropylbenzodoxazole-6-carboxamide	-6.76		
	(2-(Furan-2-yl)benzodoxazol-6-yl)(pyrrolidin-1-yl) methanone	-6.2		
	(2-(Furan-2-yl)benzodoxazol-6-yl)(piperidin-1-yl) methanone	-6.85		
	(2-(Furan-2-yl)benzodoxazol-6-yl)(morpholino) methanone	-6.99		
	N,Ndimethyl-2-(thiophen-2-yl)benzodoxazole-6- carboxamide	-6.66		
	N,N-2-(thiophen-2-yl)benzodoxazole-6- carboxamide	-6.89		
	N,Ndipropyl-2-(thiophen-2-yl)benzodoxazole-6- carboxamide	-6.71		

	Pyrrolidin-1-yl(2-(thiophen-2-yl)benzodoxazol-6- yl)methanone	-6.62		
	Piperidin-1-yl(2-(thiophen-2-yl)benzodoxazol-6-yl) methanone	-6.89		
	Morpholino(2-(thiophen-2-yl)benzodoxazol-6-yl) methanone	-7.1		
	Donepezil	-6.49		
	TAC	-5.15		
4EY6	38 compound	-9.57	AutoDock4	[62]
	42 compound	-8.95		
	Galantamine	-7.91		
1OCE	Citrinin hydrate	-6.61	MOE	[63]
	Citrinin	-6.62		
	Dicitrinol A	-8.53		
	Penicitrinol B	-9.5		
	Terrequinone A	-7.34		
	Ochrindole D	-8.11		
	Varioxiranol G	-9.76		
	Dihydrocitrinone	-6.48		
	Asterriquinone SU-5228	-8.25		
	Asterriquinone CT5	-8.02		
	Donepezil	-8.04		
	Co-crystallized inhibitor	-7.89		
4EY6	Galantamine	-9.9	AutoDock4	[64]
	ZINC16951574 (LMQC2)	-9.2		
	ZINC8342556 (LMQC5)	-10		
1EA5	Methyl dehydroabietate	-10.1	Vina	[65]
	Mintketone	-9.1		
	Sclareol	-9.1		
	T-Muurolol	-8.7		
4EY6	Dolutegravir	-8.35	Glide	[66]
	Exisulind	-10.21		
	Tretinoin	-10.02		
	Rebamipide	-11.67		
	Loracarbef	-10.17		
	Ondansetron	-7.23		
	Ramosetron	-5.91		
	Diflunisal	-7.68		

	Chlornaphazine	-6.18		
	Caroxazone	-6.16		
	Safinamide	-		
	Galantamine	-11.54		
4EY5	2	−12.10	AutoDock4	[67]
	4	−12.00		
	5	−12.20		
	6	−12.31		
	8	−12.19		
	9	−12.47		
	10	−12.75		
	Donepezil	−12.42		
4EY6	Positive control	57.97	GOLD	[68]
	Ismine	56.26		
	Kirkine	55.75		
	Maritidine	58.69		
	Tazettine	51.35		
	Anhydrolycorine	60.08		
	Homolycorine	55.13		
	Narwedine	52.79		
	Galanthindole	59.74		
	Ilc	-6.54	MOE	[69]
	Ilf	-6.01		
	Ilg	-6.23		
	Ill	-6.19		
	Galantamina	-9.28		
4EY7	€-1-(2-(Dimethylamino)-4-methylthiazol-5-yl)-3- (thiophen-2-yl)prop-2-en-1-one	-7.881	Schrödinger suite	[70]
	€-3-(4-Bromophenyl)-1-(2-(dimethylamino)-4- methylthiazol-5-yl)prop-2-en-1-one	-7.206		
4EY6	Galantamine	-7.07	MOE	[71]
4EY6	2-((5-(5-Bromobenzofuran-2-yl)-4-(3,4-dichlorophenyl)-4H-1,2,4-triazol-3-yl) thio)-N-(2,5-dimethoxyphenyl)acetamide	-9.34		
4EY7	Hispolon	-20.189	BIOVIA Software	[72]
	2-hydroxy hispolon	-18.029		

	2-hydroxy hispolon monomethyl ether	-20.356	Discovery Studio	
	Phenylethanoid glycosides of samioside	-9.969	MOE	[73]
	Forsythoside B	-8.804		
	Mol 16	-12.743	Glide	[74]
	Mol-14	-11.619		
	Mol-9	-10.237		
	Donepezil	-5.552		
	Rivastigmina	-8.98		
	Tacrina	-9.328		
	Galantamina	-9.268		
4EY7	Analog 1	−18.4	ICM Pro Molsoft	[75]
	Analog 2	−17.63		
	Analog 3	−17.24		
	Analog 4	−12.67		
	Analog 5	−16.24		
	Analog 6	−15.57		
	Analog 7	−16.31		
	Analog 8	−14.18		
	Analog 9	−15.28		
	Analog 10	−14.98		
	Analog 11	−15.38		
	Analog 12	−13.73		
	Donepezil	−17.70		
4EY6	Kushenol I	-8.9	Vina	[76]
	Kurarinone	-8.5		
	Sophoraflavanone G	-8.5		
	Isokurarinone	-9.1		
	Kushenol E	-9.3		
	Galantamine	-9.1		
4EY7	Donepezil	–15.50	MOE	[77]
	Compound 10	–14.87		
	Compound 16	–14.85		
	Compound 23	–13.85		
	Compound 31	–13.80		
	Compound 13	–13.34		
4EY7	€-1,3-Bis(4-(benzyloxy)phenyl)prop-2-en-1-one	-12		[78]

	€-1-(4-(Benzyloxy)phenyl)-3-(4-iodophenyl)prop-2-en-1-one	-11.6	Biovia Discovery Studio	
	E)-1-(4-(Benzyloxy)phenyl)-3-(p-tolyl)prop-2-en-1-one	-11.8		
4EY6	Luteolina	-11 a -12.5	Vina	[79]
4EY7	Catequina	-11 a -12.5		
4EY7	Genisteína, Tricina, Taxifolina, Crisoeriol	-11 a -12.5		
4EY7	Galantamina	-9		
4EY7	Donepezil	-8.7		
4EY7	Donepezil	-18.909	Schrödinger suite	[80]
	(+)-Elaeocarpine	-10.213		
	Genistein	-8.592		
	Apigenin 7-glucoside	-8.192		
	Verimol A	-8.185		
	Xanthyletine	-8.108		
4EY7	Viridifloral	-10.2	Vina	[81]
	2-Methyl-3-glucosyloxy-5-isopropyl phenol	-10		
	Alpha-Cucurmene	-9.9		
	Sterol	-9.8		
	Galantamine (control)	-8.9		
4EY7	Ethyl 2-((4-chlorobenzoyl)oxy)-5,6,7,8-tetrahydro-4H-cyclohepta bthiophene-3-carboxylate	-14.11	MOE	[82]
	2,3-Diphenyl-2,3,6,7,8,9-hexahydro-4H,5H-cyclohepta4,5thieno3,2-e1,3oxazin-4-one	-14.31		[82]
	2-(4-Chlorophenyl)-3-phenyl-2,3,6,7,8,9-hexahydro-4H,5H-cyclohepta4,5thieno3,2-e1,-3oxazin-4-on	-14.68		[82]
4M0E	Quinic acid	-11.75	FlexX	[83]
	Tannic acid	0		
	Quercitrin	-28.6		

	Protocatechuic acid	-22.05		
	Gallic acid	-22.97		
	Cynaroside	-21.45		
	Cosmosiin	-23.01		
	Catechin	-36.48		
	Luteolin	-27.57		
	Hesperidin	-25.18		
	Epgallocatechin gallate	-25.92		
	Isoquercitrin	-28.59		
	p-Coumaric acid	-23.19		
	Nicotiflorin	-30.03		
	Miquelianin	-26.6		
	Apigenin	-28.12		
	Naringenin	-29.21		
	Galantamine	-21.2		
4M0E	Ethyl 4-((3-cyano-4-(furan-2-yl)-6-phenylpyridin-2-yl)oxy)butanoate	-7.9	Glide	[84]
	Ethyl 4-((3-cyano-4-(furan-2-yl)-6-(p-tolyl)pyridin-2-yl)oxy)butanoate (2)	-7.7		
	Ethyl 4-((4-(4-chlorophenyl)-3-cyano-6-(p-tolyl)pyridin-2-yl)oxy)butanoate	-8.9		
	ethyl 4-((3-cyano-4-(4-isopropylphenyl)-6-(p-tolyl)pyridin-2-yl)oxy)butanoate	-9.7		
	Ethyl 4-((3-cyano-4-(4-methoxyphenyl)-6-phenylpyridin-2-yl)oxy)butanoate	-8.8		
	Ethyl 4-((3-cyano-4-(4-methoxyphenyl)-6-phenylpyridin-2-yl)oxy)butanoate	-7.2		
	Ethyl 4-((3-cyano-4,6-diphenylpyridin-2-yl)oxy)butanoate	-9.2		
	Galantamine	-7		
4EY7	2-(((4-Benzylcyclohexyl)ethylamino)methyl)-5-(benzyloxy)- 1-methylpyridin-4(1H)-one	-10.3	NA	[85]
	2-((1-(4-Methoxyphenethyl)piperidin-4-	-10.5	NA	[85]

	ylamino)methyl)- 5-hydroxy-1-methylpyridin-4(1H)-one			
5HFA	Δ8-THC	-5.5	Discovery Studio	[86]
	CBG	-5.2		
	CBGA	-4.9		
	CBT	-5.4		
	CBDV	-5.9		
	Galantamine	-8.2		
6WO4	2-(1H-Indol-3-yl)-N-propyl-1H-benzodimidazole-5-carboxamide	-10.83	Smina	[87]
	N-Allyl-2-(1H-indol-3-yl)-1H-benzodimidazole-5-carboxamide	-10.95		
	2-(1H-Indol-3-yl)-N-isopropyl-1H-benzodimidazole-5-carboxamide	-8.55		
	N-Cyclopropyl-2-(1H-indol-3-yl)-1H-benzodimidazole-5-carboxamide	-10.83		
	N-Butyl-2-(1H-indol-3-yl)-1H-benzodimidazole-5-carboxamide	-11.02		
	2-(1H-Indol-3-yl)-N-isobutyl-1H-benzodimidazole-5-carboxamide	-11.02		
	N-Cyclohexyl-2-(1H-indol-3-yl)-1H-benzodimidazole-5-carboxamide	-10.35		
	2-(1H-Indol-3-yl)-N-phenyl-1H-benzodimidazole-5-carboxamide	-10.63		
	2-(1H-Indol-3-yl)-N-(m-tolyl)-1H-benzodimidazole-5-carboxamide	-10.8		
	2-(1H-Indol-3-yl)-N-(4-methoxyphenyl)-1H-benzodimidazole-5-carboxamide	-10.75		
	N-Benzyl-2-(1H-indol-3-yl)-1H-benzodimidazole-5-carboxamide	-10.63		
	2-(1H-Indol-3-yl)-N-phenethyl-1H-benzodimidazole-5-carboxamide	-8.93		
	N-(3,4-Dimethoxyphenethyl)-2-(1H-indol-3-yl)-1H-benzodimidazole-5-carboxamide	-11.62		
	Tacrine	-8.55		

1EVE	4-Methyl-2-oxo-2H-chromen-7-yl Thiazol-2-ylglycinate	-27.99	Biovia Discovery Studio	[88]
	4-methyl-2-oxo-2H-chromen-7-yl Benzodthiazol-2-ylglycinate	-30.15		
	4-Methyl-2-oxo-2H-chromen-7-yl (4-Methylthiazol-2-yl)glycinate	-32.01		
	4-Methyl-2-oxo-2H-chromen-7-yl (5-Nitrobenzodthiazol-2-yl)glycinate	-29.49		
	4,5-Dimethyl-2-oxo-2H-chromen-7-yl Thiazol-2-ylglycinate	-25.92		
	4, 5-Dimethyl-2-oxo-2H-chromen-7-yl Benzodthiazol-2-ylglycinate	-25.61		
	4,5-Dimethyl-2-oxo-2H-chromen-7-yl (4-methylthiazol-2-yl)Glycinate	-22.02		
	4,5-Dimethyl-2-oxo-2H-chromen-7-yl (5-nitrobenzodthiazol-2-yl)Glycinate	-24.47		
	4-(Chloromethyl)-2-oxo-2H-chromen-7-yl Thiazol-2-ylglycinate	-31.15		
	4-(Chloromethyl)-2-oxo-2H-chromen-7-yl (5-Nitrobenzodthiazol-2-yl)glycinate	-29.74		
	4-(Chloromethyl)-2-oxo-2H-chromen-7-yl (5-Nitrothiazol-2-yl)glycinate	-32.219		
6O4X	Quercetin	-10.61	Glide	[89]
	Isorhamnetin	-10.2		
	Ginkgotoxin	-9.91		
	Kaempferol	-9.76		
	Quercetin-3-β-glucoside	-8.96		
	Gingkolide B	-8.86		

	Rutin	-8.68		
	Quercitrin	-8.09		
	Galantamine	-8.02		
	Dihydrotanshinone I	-7.05		
	Bilobalide	-4.83		
	Gingkgolide C	-4.8		
	Ginkgolide A	-4.71		
4M0E	Donepezil	-45.18	CDOCKER	[90]
	DHI	-38.92		
	5,7-dihydroxy-2-phenyl-4H-chrome-4-one	-35.28		
	4-phenyl-2-(pyridin-3-yl)quinazoline	-32.65		
4PQE	Donepezil	-8.6	Biovia Discovery	[91]
	Curcumin	-7.5		
	Gedunin	-8.7		
	Quercetin	-7.5		
	Resveratrol	-7		
	Nobiletin	-6.9		
	Fisetin	-8.2		
	Berberine	-7.7		
6O4W	1-(4-(Diisopropylamino)but-2-yn-1-yl)-3,7-dimethyl-3,7-dihydro-1H-purine-2,6-dione	-15.207	Glide	[92]
	Donepezil	-14.817		
	1-(4-(Azocan-1-yl)but-2-yn-1-yl)-3,7-dimethyl-3,7-dihydro-1H-purine-2,6-dione	-14.742		
	7-(4-(Diisopropylamino)but-2-yn-1-yl)-1,3-dimethyl-3,7-dihydro-1H-purine-2,6-dione	-14.733		
	3,7-Dimethyl-1-(4-(4-(2-(pyrrolidin-1-yl)ethyl)piperazin-1-yl)but-2-yn-1-yl)-3,7- dihydro-1H-purine-2,6-dione hydrate	-14.59		
	7-(4-(Azocan-1-yl)but-2-yn-1-yl)-1,3-dimethyl-3,7-dihydro-1H-purine-2,6-dione	-13.84		
	1,3,7-Trimethyl-8-(3-(4-(2-(pyrrolidin-1-yl)ethyl)piperazin-1-yl)prop-1-yn-1-yl) -3,7-dihydro-1H-purine-2,6-dione hydrate	-12.86		

	1,3,7-Trimethyl-8-(3-(4-methylpiperidin-1-yl)prop-1-yn-1-yl)-3,7-dihydro-1Hpurine-2,6-dione	-12.693		
	58	-12.432		
	8-(3-(Diisopropylamino)prop-1-yn-1-yl)-1,3,7-trimethyl-3,7-dihydro-1H-purine2,6-dione	-11.833		
	8-(3-(Azocan-1-yl)prop-1-yn-1-yl)-1,3,7-trimethyl-3,7-dihydro-1H-purine-2,6-dione	-10.691		
	1,3-Dimethyl-7-(4-(4-(2-(pyrrolidin-1-yl)ethyl)piperazin-1-yl)but-2-yn-1-yl)-3,7- dihydro-1H-purine-2,6-dione	-10.542		
	8-(3-(Dicyclohexylamino)prop-1-yn-1-yl)-1,3,7-trimethyl-3,7-dihydro-1H-purine2,6-dione	-9.562		
	8-(3-(Azepan-1-yl)prop-1-yn-1-yl)-1,3,7-trimethyl-3,7-dihydro-1H-purine-2,6-dione	-9.561		
	8-(Furan-3-yl)-1,3,7-trimethyl-3,7-dihydro-1H-purine-2,6-dione	-7.931		
	8-(1-(2-Hydroxyethyl)-1H-1,2,3-triazol-4-yl)-1,3,7-trimethyl-3,7-dihydro-1H-purine-2,6-dione	-7.712		
	8-(1-Butyl-1H-1,2,3-triazol-4-yl)-1,3,7-trimethyl-3,7-dihydro-1H-purine-2,6-dione	-7.053		
	8-Ethynyl-1,3,7-trimethyl-3,7-dihydro-1H-purine-2,6-dione {8-Ethynylcaffeine}	-6.512		
6O4W	Caffeine	-6.284		
	Compound I	−8.1	Vina	[93]
	Compound II	−8.5		
	Compound III	−8.9		
	Compound IV	−9.2		
	Donepezil (ref)	−11.1		
73EH	N,N0 –(1,3-phenylene)bis(3,4,5-trimethoxybenzamide)	-9.8	Vina	[94]
	N,N0 –(1,3-phenylene)bis(3,5-dimethoxybenzamide)	-10.3		

	N,N0 –(1,3-phenylene)bis(3,4-dimethoxybenzamide)	-10.4		
	N,N0 –(1,3-phenylene)bis(3-methoxybenzamide)	-11.2		
	N,N0 –(1,4-phenylene)bis(3,4,5-trimethoxybenzamide)	-10		
	N,N0 –(1,4-phenylene)bis(3,5-dimethoxybenzamide)	-10.7		
	N,N0 –(1,4-phenylene)bis(3,4-dimethoxybenzamide)	-10.8		
	N,N0 –(1,4-phenylene)bis(3-methoxybenzamide)	-11.2		
	3,4,5-trimethoxy-N-(3-nitrophenyl)benzamide	-9.4		
	3,4,5-trimethoxy-N-(3,4,5-trimethoxyphenyl)benzamide	-8.8		
	3,4,5-trimethoxy-N-(3,4,5-trimethoxybenzyl)benzamide	-8.7		
	Donepezil	-11.6		
	Tacrine	-8.9		
	Quercetin	-9.3		
4M0E	Dihydrotanshinone I	-15	Vina	[95]
4EY6	Galanthamine	-14.2		
4EY5	Huperzine A	-14.2		
7D9Q	H1R	-18.8		
7D9O	H0L	-18.3		
7XN1	Tacrine	-12.1		
4EY7	Donezepil	-18.1		
7D9P	H0R	-18.4		
1EVE	Compound 2	5.8942	Surflex-Dock	[96]
	A1	6.7088		
4EY5	HupA (1)	–12.17		
	C17H22N2O	-11.436	Glide	[96]
	C17H22N2O	-11.107		
	C17H20N2O	-10.792		
	C13H20N2O	-10.672		
	C15H19N2O	-10.12		
1C2O	1	–8.82	AutoDock4	[97]
	1f	–10.17		

	TAC*	–7.10		
4EY5	6a	−5.68	AutoDock4	[98]
	6b	−7.35		
	6c	−6.32		
	Thioflavin T (control)	−8.29		
1EVE	BOP-1	–10.75	AutoDock4	[99]
	BOP-8	–10.26		
4PQE	3a	−8.37	Achilles Docking Server	[100]
	3b	−8.50		
	3c	−7.67		
	4	−8.07		
	6	−6.90		
4PQE	Beta-carotene	-4.9	Vina	[108]
	Stigma sterol	-5.8		
	Quercetin	-6.7		
	Xanthophylls	-7.2		
	Beta-sitosterol	-10.56		
	Dihydroquercetin	-11.8		
4EY7	Tumulosis acid	-46.26	CDOCKER	[108]
	Lanosteryl acetate	-45.83		
	Pachymic Acid	-39.02		
	Alisol C monoacetate	-35.74		
	Tormentic acid	-33.34		
	Donepezil	-31.26		
6O69	Aposcopolamine	-4.4377	MOE	[109]
	Inermin	-4.4852		
	Stigmasterol	-5.1831		
4EY7	Fangchinoline	55.9794	CDOCKER	[110]
	Dauricine	84.4849		
	Baicalein	41.0117		
	Chrysin	40.5337		
	Oroxylin A	46.1022		
	Quercetin	43.9071		
	Wogonin	41.485		
4M0E	Kaempferol 3-o-robinobioside	-8.26	MOE	[111]
	Kaempferol 3-o-β-D-glucoside	-7.64		
3LII	1-Heneicosanol	-85.99	iGEMDOCK	[112]
	N-Nonadecanol-1	-81.06		
	Cholesta-4,6-dien-3-ol (3beta)	-92.38		

	Di-n-octyl phthalate	-91.43		
	7,9-Di-tert-butyl-1-oxaspiro(4,5)deca-6,9-diene-2,8-dione	-87.6		
	6-Undecyl-5,6-dihydro-2H-pyran-2-one	-87.31		
	Phenol, 2,4-di-t-butyl-6-nitro	-86.65		
4EY7	Fangchinoline	-7.11	MOE	[113]
4EY8	Cyclanoline	-7		
1GQS	PJ5	-5.4	Vina	[114]
	PJ13	-9.3		
	PJ15	-7.8		
6O4W	PJ5	-3.7		
	PJ13	-8.6		
	PJ15	-8.7		
6EUC	Pyriproxyfen S	-9	Vina	[115]
	Pyriproxyfen R	-8.7		
1C2B	Acetylthiocholine	-54.54	DSHC	[116]
	5-Methylguaiacol	-28.36		
	p-Cresol	-19.16		
	Guaiacol	-24.07		
	o-Cresol	-27.06		
	2,4-Dimethylphenol	-31.24		
	m-Cresol	-24.81		
	Phenol	-16.57		
	4-Methylguaiacol	-26.92		
NA	Rosmarinic acid	-41.82	DOCK	[117]
	Salvigenin	-31.14		
	Salvianolic acid B	-15.94		
	Salvianolic acid A	-41.48		
	Tanshinone IIA	-20.24		
	Dihydrotanshinone I	-52.44		
	Tanshinone I	-30.11		
	Carnosic acid	-22.07		
	Carnosol	-42.14		
	Cryptotanshinone	-26.53		
	Danshensu sodium salt	-13.95		
NA	Paraoxon-CB7	-35.1	Vina + Umbrella	[118]
	Atropine-CB7	-41.72		
	K027-CB7 oxmate	-53.26		

	K027-CB7 amide	-18.46	Sampling simulation	
NA	1a	-12.01	Vina	[119]
	1b	-11.97		
	1c	-12.94		
	1d	-12.26		
	1e	-11.38		
	1f	-11.39		
	1g	-12.58		
	1h	-12.24		
	2a	-11.88		
	2b	-12.1		
	2c	-12.75		
	2d	-11.92		
	2e	-13.02		
	2f	-11.64		
	2g	-11.56		
	2h	-12.89		
4PQE	4a	-7.3	Vina	[120]
	4b	-7.2		
	4c	-6.9		
	4d	-7.2		
	4e	-7.3		
6H12	5-N-methylmaytenine	-10.5	Vina	[120]
	Stepharine	-10.3		
4M0E	Hybrid G	<-7.1		
	Dihydrotanshinone	<7.1		
1DX6	B3	-17.55	FlexX BioSolvelT Leadlt	[121]
	D5	-21.8		
	D6	-20.39		
4EY7	Asiatic acid	-10.27	AutoDock4	[122]
	Madecassic acid	-8.7		
	Madecassoside	81.61		
	Asiaticoside	41.72		
	Eserine (Control)	-9.4		
4BDT	5	-10.34	AutoDock4	[123]
	6	-10.63		
	7	-9.55		

	8	-9.35		
	9	-10.27		
	10	-10.29		
	11	-9.47		
	12	-10.15		
	13	-11.51		
NA	1m	−7.7	Vina	[124]
	1p	−9.1		
	2a	−8.5		
	2e	−8.0		
	3b	−8.0		
1ACJ	Geraniol (CAS)	−5.6	Vina	[125]
	Geraniol (PAS)	−6.8		[125]
	Acetylcholine	−4.1		[125]
1OCE	Piperine	−7.01	Discovery Studio	[126]
	Sesamin	−25.80		
1H23	Epicatechin	−8.0 a −11.4	Vina	[127]
	Procyanidin B2	−8.0 a −11.4		
5FUM	Biatractylenolide II	−8.2	Vina	[128]
1QON	(+)-Cymbodiacetal	−9.407	Schrödinger Suite	[129]
	Proximadiol	−8.253		
	Geranylacetone	−8.177		
	Rutin	−8.148		
	Ligando estándar (1QON)	−15.402		
1ACJ	Geraniol	−6.8	Vina	[130]
	Acetylcholine	NA		
4EY7	Vanilic acid	-6.8	AutoDock4	[131]
	Quercitrin	-8.8		
4BDT	SG4	-2.753	Schrödinger	[132]
1ACL	(E)-2-(2-(2-((1H-benzodimidazol-2-yl)thio)-1-(3,4-dichlorophenyl)ethylidene) hydrazinyl)-4-(3,4-dichlorophenyl)thiazole	-11.23	MOE	[133]
	(E)-4-(2-(2-(2-((1H-benzodimidazol-2-yl)thio)-1-(2-nitrophenyl)ethylidene) hydrazinyl) thiazol-4-yl)phenol	-10.7		
	(E)-2-(2-(2-((1H-benzodimidazol-2-yl)thio)-1-(2-nitrophenyl)ethylidene)	-9.33		

	hydrazinyl)-4-(3,4-dichlorophenyl)thiazole			
1ACL	I-4-(2-(2-(2-((1H-Benzodimidazol-2-yl)thio)-1-(p-tolyl)ethylidene)hydrazinyl) Oxa-zol-4-yl)phenol	-12.37	Vina	[133]
	€-2-(2-(2-((1H-Benzodimidazol-2-yl)thio)-1-(p-tolyl)ethylidene)hydrazinyl)-4- (2,4-dichlorophenyl)oxazole	-11.97		
	€-2-(2-(2-((1H-Benzodimidazol-2-yl)thio)-1-(p-tolyl)ethylidene)hydrazinyl)-4- (3,4-dichlorophenyl)oxazole	-10.12		
1EVE	Lactucin	-37.64	Schrödinger Suite	[134]
	Lactucopicrin	-47.9		
4EY6	((1S,9R)-11-(2E)-3-phenylprop-2-enoyl-7,11-diazatricyc lotrideca-2,4-dien-6-one)	-44.44	GOLD	[135]
	(3-methyl-10 ,20 ,3,4,5,10- hexahydro-2H-spiroazepino 3,4-b indole-1,30 -indol-20 -one)	-55.84		
	(N-(4-methylphenyl)- 2-{5-oxo-1H,2H,3H,4H,5H,6H-azepino 4,5-bindol-4-yl} acetamide)	-59.14		
	(N-{(1S,9aR)- octahydro-1H-quinolizin-1-ylmethyl}-2-(6-fluoro-1H-indol-1-yl)acetamide)	-65.81		
	(60 -methyl-1-(prop-2-en-1- yl)-1,2,20 ,30 ,40 ,90 -hexahydrospiroindole-3,10 -pyrido 3,4-bindol-2-one)	-60.2		
6XYU	6-methyl-2-oxo-N-(5-((4,11,11-trimethyl-1,2,3,4-tetrahydro-1,4-methanoacridin-9-yl)amino)pentyl)-2H-chromene-3-carboxamide	-37.48	AutoDock4	[136]
	7-methyl-2-oxo-N-(6-((4,11,11-trimethyl-1,2,3,4-tetrahydro-1,4-methanoacridin-9-yl)amino)hexyl)-2H-chromene-3-carboxamide	-40.43		
1F8U	Asterric acid	-7.89	AutoDock4	[137]

	Methyl asterrate	-9.72		
	Ethyl asterrate	-9.74		
1ACL	(.E)-N-(4,5-dichloro-2-nitrophenyl)-2-(1-(3-hydroxy-2-nitrophenyl)-2-((5-(4-nitrophenyl)-4H-1,2,4-triazol-3-yl)thio)ethylidene)hydrazine-1-carbothioamide	-12.13	MOE	[138]
	(.E)-N-(4,5-dichloro-2-nitrophenyl)-2-(1-(2-hydroxyphenyl)-2-((5-(4-nitrophenyl)- 4H-1,2,4-triazol-3-yl)thio)ethylidene)hydrazine-1-carbothioamide	-11.37		
	(.E)-2-(1-(3,4-dichlorophenyl)-2-((5-(4-nitrophenyl)-4H-1,2,4-triazol-3-yl)thio)ethylidene)-N-(3-nitrophenyl)hydrazine-1-carbothioamide	-10.63		
1ACL	Baicalein	-33.26	CDOCKER	[139]
1EEA	Glucolimnanthin	-9.2	Vina	[140]
	Glucosinalbin	-9		
	IMG	-9.6		
	Glucoalyssin	-7.9		
	Methoxyglucobrassicin	-9.5		
	4-methoxyindolyl-3-hexylhydroxyglucosinolate	-8.9		
	pent-4- enylglucosinolate	-8.2		
	indolyl-3-hexyl-4-methylcyclohexaneglucosinolate	-9.6		
	1-isothiocyanato-9-methanesulfinylnonane	-5.8		
6H12	Lindoldhamine isomer	-114.6	Schrödinger Suite	[141]
	Sthepharine	-68.87		
	Palmatine	-74.87		
	5-N-methylmaytenine	-82.01		
	N-trans-feruloyltyramine	-68.43		
	Neostigmine	-61.71		
2ACK	Tumulosic acid	-7.77	Vina	[142]
	Polyporenic acid C	-7.88		
	3-Epidehydrotumulosic acid	-7.37		
	Pachymic acid	-8.01		
	Dehydro-trametenolic acid	-6.92		

	Huperzine A	-7.51		
4M0E	Co-crystallized ligand	-18.6	Vina	[143]
	Marrubin	-19.34		
7E3H	Rivastigmine	-7.8	AutoDock4	[144]
	(-)- Epicatechin gallate CID: 107905	-12.2		
	4-((40 – (Aminomethyl)- 1,10 - biphenyl-3- yl)oxy)pyrimidine2-carbonitrile CID: 12000657	-11.6		
4M0E	Neostigmine	-7.5	Vina	[145]
	14-methyl-16-azabicyclo10.3.1hexadeca-1(15),12(16),13-triene	-7.1		
4EY7	Chrysin (or 5,7-dihydroxyflavo	-10.4	Vina	[146]
	Icariin	-8.2		
	6-hydroxyflavone	-10.2		
	5-hydroxyflavone	-10.3		
	7-hydroxyflavone	-10.4		
	trihydroxyethylrutin	-9		
	Baicalein (or 5, 6, 7-trihydroxyflav	-10.6		
	3′,5′-dihydroxyflavo	-10.3		
	3′-hydroxy-b-naphthoflavo	-12.4		
	3 0 -hydroxy-a-naphthoflavone	-11.2		
	5-hydroxy-3′-methoxyflavone	-10.5		
	4′-5-dihydroxy-7-methoxyflavone	-10.4		
	7,3′-dihydroxyflavone	-10.5		
	5,3′-dihydroxyflavone	-10.3		
	5-hydroxy-7-((3-methylbenzyl)oxy)-2- phenyl-4h-chromen-4-one	-11.6		
	3′-hydroxy-6-methylflavone	-10.9		
	6-hydroxy-4′-methylflavone	-10.2		
	7-hydroxy-3′,4′,5′-trimethoxy-α-naphthoflavone	-10.4		
	Diosmin	-10.9		
	Myricitrin dihydrate	-8.1		
4EY4	6-Methyl-6H-thiazolo5,4-eindazol-2-amine	-7.5	Vina	[147]
	6-Ethyl-6H-thiazolo5,4-eindazol-2-amine	-7.9		
	1-Methyl-1H-thiazolo4,5-gindazol-7-amine	-7.5		

	1-Ethyl-1H-thiazolo4,5-gindazol-7-amine	-7.6		
	8-Chloro-6-methyl-6H-thiazolo5,4-eindazol-2-amine	-7.9		
	2-Chloro-N-(6-methyl-6H-thiazolo5,4-eindazol-2-yl)-acetamide	-7.4		
	2-Chloro-N-(6-ethyl-6H-thiazolo5,4-eindazol-2-yl)-acetamide	-7.7		
	2-Chloro-N-(8-chloro-6-methyl-6H-thiazolo5,4-e-indazol-2-yl)acetamide	-8		
	2-Azido-N-(6-methyl-6H-thiazolo5,4-eindazol-2-yl)-acetamide	-8.1		
	2-Azido-N-(6-ethyl-6H-thiazolo5,4-eindazol-2-yl)-acetamide	-8.3		
	2-Azido-N-(8-chloro-6-methyl-6H-thiazolo5,4-e-indazol-2-yl)acetamide	-8.7		
	T145a[1]	-12.7		
	T134a[1]	-11.8		
	T158a[1]	-11.7		
	T121a[1]	-11.5		
	T128a[1]	-11.3		
	T145b[2]	-12.3		
	T158b[2]	-11.6		
	T1428[2]	-11.3		
	T127b[2]	-10.8		
	T142b[2]	-10.8		
	T145c[3]	-12.8		
	T158c[3]	-12.1		
	T134c[3]	-12		
	T144c[3]	-11.5		
	T111c[3]	-11.1		
4EY7	Rivastigmine	-8.342	Glide	[148]
	Col_trp rigid R,S	-12.652		
	Col_phe non-rigid (S,S)	-11.097		
	Col_arg rigid (R,S)	-11.027		
	Col_arg rigid (R,R)	-11.011		
	Col_tyr non-rigid (S,R)	-11.415		

4EY7	M2	-13	Biovia Discovery studio	[149]
	M1	-12.6		
	M6	-12.4		
	Donepezil	-10.8		
	Rugin	-17.023		
6CQV	HI6	-4.39	Vina	[150]
4M0E	Dihydrotanshinone I	-8.24		
4EY6	Galantamine	-9.61		
4EY5	Huperzine A	-10.54		
6O50	EBW	-11.11		
4M0F	Territrem B	-11.29		
7D9Q	H1R	-11.7		
7D90	H0L	-11.77		
4EY7	Donepezil	-11.94		
7D9P	H0R	-12.44		
1EVE	Donepezil (Reference drug)	-12.74	ArgusLab 4.0	[151]
1EVE	2-Chloro-N –(2- (piperidine-1-yl) ethyl) benzamide	-9.83		
1EVE	3-Chloro-N-(2- (piperidine-1-yl) ethyl) benzamide	-9.92		
1EVE	4-Chloro-N- (2- (piperidine-1-yl)ethyl) benzamide	-10.17		
1EVE	2-Fluoro-N- (2- (piperidine-1-yl) ethyl) benzamide	-9.76		
1EVE	3-Fluoro-N-(2- (piperidine-1-yl) ethyl) benzamide	-9.63		
1EVE	4-Fluoro-N-(2- (piperidine-1-yl) ethyl) benzamide	-9.53		
1EVE	2-Nitro-N-(2-(piperidine-1-yl)ethyl)benzamide	-9.45		
1EVE	3-Nitro-N-(2-(piperidine-1-yl)ethyl)benzamide	-8.92		
1EVE	4-Nitro-N-(2-(piperidine-1-yl)ethyl)benzamide	-9.14		
1EVE	2-Methoxy-N-(2- (piperidine-1-yl) ethyl) benzamide	-9.26		
1EVE	3-Methoxy-N-(2- (piperidine-1-yl) ethyl) benzamide	-8.96		
1EVE	4-Methoxy-N-(2- (piperidine-1-yl) ethyl) benzamide	-9.1		

1C2B	Arcabose (control)	-4.8	Vina	[152]
1C2B	Kaempferol	-8.6		
1C2B	Quercetin	-6.9		
1C2B	Emodin	-7.7		
1C2B	Chrysophanol	-3.8		
1C2B	Donepezil (Reference drug)	-11.7		
4M0E	Donepezil	-8.271	Schrödinger suite	[153]
	HIT 1	-12.096		
	HIT 2	-11.666		
	HIT 3	-11.269		
	HIT 4	-11.174		
	HIT 5	-10.613		
	HIT 6	-10.525		
	HIT 7	-10.512		
	HIT 8	-10.402		
	HIT 9	-10.391		
	HIT 10	-8.271		
4EY7	8-acetil-7-hidroxi-4-metilcumarina	-76.32	Schrödinger suite	[154]
	4,7-dimetil-5-hidroxicumarina	-70.12		
7D9P	HOR	-10.6	Vina	[155]
	Biflavanone	-13.1		
	Calomenalol J	-12		
4EY7	Khusilol	-8.324	Biovia Discovery Studio	[156]
	Ferulic acid methyl ester	-7.533		
	3-Pinanone	-7.226		
	Borneol	-6.524		
	Borneol acetate	-7.834		
	Caffeic acid methyl ester	-7.489		
	Camphor	-6.983		
	Caryophyllene oxide	-8.564		
	Cis-Linalool oxide	-6.551		
	Dienestrol	-9.494		
	Hexanal	-4.576		
	Honokiol	-9.442		
	Limonene	-7		
	Magnaldehyde D	-9.39		
	Magnolol	-9.825		
	Obovatol	-9.827		
	Phenol	-5.25		
	Randaiol	-8.558		

	α-Eudesmol	-9.856		
	α-Terpineol	-7.086		
	β-Eudesmol	-8.482		
	γ-Eudesmol	-9.668		
	γ-Gurjunene epoxide	-7.956		
	C6R	-		
	E20	-12.2		
	PDB	-		
	Protoberberine	-11.2	Schrodinger suite	[156]
	Protopine	-10.864		
	Codeine	-10.204		
	Donepezil	-10.171		
3LII	VPGYPFLPI	-974	ClusPro y PyMol	[157]
	KSPCVFILDQKKRL	-792.2		
1O86	BXZ-LysR	-12.18	AutoDock4	[158]
	BXZ-ArgS	-11.95		
	BXZ-OrnR	-11.48		
	BXZ-PheS	-11.1		
	BXZ-HisR	-10.46		
4EY7	Aromadendrene	-8.8	Vina	[159]
	Camphene	-6.5		
	Caryophyllene Oxide	-8.6		
	Copaene	-8.6		
	Cubenol	-8.7		
	Epi- γ-eudesmol	-9.2		
	Eucalyptol	-6.9		
	Farnesol	-8.6		
	Linalool	-6.5		
	Nerolidol	-8.3		
	Spathulenol	-8.4		
	Valencene	-8.8		
	α-Bisabolol	-8.7		
	α-Bisabolol Oxide A	-9.5		
	α-Bisabolol Oxide B	-9.1		
	α-Cadinene	-9.1		
	α-Humulene	-7.9		
	α-Pinene	-6.9		
	α-Thujene	-6.8		
	β-Caryophyllene	-8.4		

	β-Elemene	-8.1		
	β-Pinene	-6.9		
	γ-Elemene	-7.9		
	γ-Muurolene	-9		
	δ-Cadinene	-9.1		
	Donepezil	-11.8		
	Galanthamine	-10.5		
	Huperzine A	-9.9		
	Neostigmine	-7.4		
	Physostigmine	-9.1		
	Pyridostigmine	-6.3		
	Rivastigmine	-8.1		
	Tacrine	-8.8		
4BDT	Donepezil (Reference drug)	-8.5	MOE	[160]
	Apigenin-7-O-glucoside	-8.83		
	α- Tocopherols	-7.93		
	Atractyol	-7.62		
	Protocatechuic acid	-7.61		
	Rhoifolin	-7.56		
	Gallic acid	-7.18		
	p-Hydroxybenzoic acid	-6.55		
	γ- Tocopherols	-6.19		
	δ- Tocopherols	-6.19		
	Ecgonine methyl ester	-5.63		
4EY6	Thiamine T	-2.465	Schrödinger suite	[161]
4EY6	Thiamine phyrophosphate TPP	0.823		
7XN1	Thiamine T	-10.735		
7XN1	Thiamine phyrophosphate TPP	-11.054		
4EY7	Aposcopolamine	-4.4377	Schrödinger suite	[162]
	Inermin	-4.4852		
	Galangin	-10.2		
	Isoliquiritigenin	-12.616		
	Naringenin	-11.043		
	Palmatoside G	-14.156		
	Quercetin	-11.709		
	Rugin	-15.943		
	Chrysin	-7.731		
	Convolvine	-2.555		

	Galangin	-8.422		
	Isoliquiritigenin	-7.524		
	Naringenin	-8.758		
	Palmatoside G	-9.839		
	Quercetin	-10.912		
	Rugin	-11.529		
6EYF	Chrysin	-6.857	Schrödinger suite	[162]
	Convolvine	-6.303		
	Galangin	-9.121		
	Isoliquiritigenin	-7.009		
	Naringenin	-7.721		
	Palmatoside G	-8.255		
	Quercetin	-8.967		
	Rugin	-13.103		
3I6Z	2-CIMHB	-11.8446	MOE	[163]
	2-CIBHB	-11.5742		

Table S2. Molecular dynamic studies about AChE.

PDB ID	Ligand	Time (ns)	Software	RMSD (Å)	Cite
4EY7	8-acetyl-7-{3-4-(3- methoxyphenyl)piperazin-1-ylpropoxy}-4-methylchromen-2-one	120	NA	~0.25	[21]
	8-acetyl-7-{4-4-(3- methoxyphenyl)piperazin-1-ylbutoxy}-4-methylchromen-2-one	120		~0.25	
4M0E	4-Amino-2-styrylquinoline	NA	Desmond	~0.27	[40]
	Bisdemethoxycurcumin	NA		~0.28	
	Donepezil (Reference drug)	NA		~0.3772	
	Apigenin-7-O-glucoside	NA		~0.3622	
	α- Tocopherols	NA		~0.2689	
	Atractyol	NA		~0.3996	
4EY7	M1	100	Desmond	~0.42	[49]
	M2	100		~0.35	
	Donepezil	100		~0.61	
4BDT	Protocatechuic acid	NA	NA	~0.4181	[65]
	Rhoifolin	NA		~0.3503	
	Gallic acid	NA		~0.3665	
	p-Hydroxybenzoic acid	NA		~0.129	
	γ- Tocopherols	NA		~0.4388	
	δ- Tocopherols	NA		~0.4388	
	Ecgonine methyl ester	NA		~0.4525	
4EY5	$C_{17}H_{22}N_2O$	100	GROMACS-2023	~0.156	[70]
	$C_{17}H_{22}N_2O$	100		~0.138	
4EY6	Galantamine	-7.07	MOE	NA	[71]
	2-((5-(5-Bromobenzofuran-2-yl)-4-(3,4-dichlorophenyl)-4H-1,2,4-triazol-3-yl) thio)-N-(2,5-dimethoxyphenyl)acetamide	-9.34		NA	
4EY7	Tumolosic acid	90	NAMD 3.0 alpha	~0.24	[72]
7D9O	H0L	50	GROMACS	~0.26	[73]
7XN1	Tacrine	50		~0.27	

4EY7	Donepezil	50		~0.28	
7D9P	H0R	50		~0.27	
7D9O	H0L	50		~0.26	
7XN1	Tacrine	50		~0.27	
4EY7	Donepezil	50		~0.28	
7D9P	H0R	50		~0.27	
4EY7	Protopine	250	Desmond v3.6	~0.0655	[78]
	Codeine	250		~0.0681	
	Donepezil	250		~0.2259	
	(E)-1,3-Bis(4-(benzyloxy)phenyl)prop-2-en-1-one	250		~0.239	
4EY7	Donepezil	50	NA	~0.29	[85]
	2-(((4-Benzylcyclohexyl)ethylamino)methyl)-5-(benzyloxy)- 1-methylpyridin-4(1H)-one	50		~0.30	
	2-((1-(4-Methoxyphenethyl)piperidin-4-ylamino)methyl)- 5-hydroxy-1-methylpyridin-4(1H)-one	50		~0.29	
4EY7	CLOZ	150	GROMACS 2023	~0.60	[88]
4M0E	Hybrid G	200	GROMACS 5.1.2	~0.25	[153]
	Galantamine	150		~0.26	
7RB5	5-Benzyl-4-(4-chlorophenyl)-2-spiro2,600 indenoquinoxalino-3-spiro3,30 -chromanonopyrrolidine	150	GROMACS	~0.25	[154]
4M0E	HIT 1	100	GROMACS 2021.6	~0.30	[155]
	HIT 2	100		~0.28	
	8-acetyl-7-{3-4-(2- methoxyphenyl)piperazin-1-ylbutoxy}-4-methylchromen-2-one	100		~0.25	
4EY7	(E)-3-(4-Bromophenyl)-1-(2-(dimethylamino)-4- methylthiazol-5-yl)prop-2-en-1-one	100	GROMACS 2024.2	~0.26	[156]

	(E)-1-(2-(Dimethylamino)-4-methylthiazol-5-yl)-3- (thiophen-2-yl)prop-2-en-1-one	100		~0.24	
7D9P	HOR	100	GROMACS	~0.19	[157]
	Biflavanone	100		~0.19	
	Calomenalol J	100		~0.20	
4EY7	Honokiol	2	GROMACS 2023.3	~0.24	[158]
	Protoberberine	2		~0.0553	
4EY7	(E)-1-(4-(Benzyloxy)phenyl)-3-(p-tolyl)prop-2-en-1-one	100	GROMACS V2022.4	~0.219	[159]
	3-(1-methylpiperidin-2-yl)phenyl N,N-diethylcarbamate	100		~0.35	
4EY6	Thiamine T	100	Desmond	~0.20	[160]
	Thiamine pyrophosphate TPP	100		~0.32	
7XN1	Thiamine T	100		~0.20	
	Thiamine pyrophosphate TPP	100		~0.25	
	E2020	100		~0.34	
1EVE	4,5-Dimethyl-2-oxo-2H-chromen-7-yl Thiazol-2-ylglycinate	50	Discovery Studio 2020	~0.32	[161]
	Dihydrotanshinone I	50		~0.22	
	Galanthamine	50		~0.27	
	Huperzine A	50		~0.25	
	H1R	5		~0.26	
6CQV	HI6	50	GROMACS 2019.6	~0.27	[164]
	Dihydrotanshinone I	50		~0.24	
	Galantamine	50		~0.25	
	Huperzine A	50		~0.26	
	EBW	50		~0.24	

	Territrem B	50		~	
	H1R	50		~	
	H0L	50		~	
	Donepezil	50		~	
	H0R	50		~	
	Dihydrotanshinone I	50		~0.22	
	Galanthamine	50		~0.27	
	Huperzine A	50		~0.25	
	H1R	50		~0.26	
